# Dapagliflozin Does Not Modulate Atherosclerosis in Mice with Insulin Resistance

**DOI:** 10.3390/ijms21239216

**Published:** 2020-12-03

**Authors:** Alida Taberner-Cortés, Ángela Vinué, Andrea Herrero-Cervera, María Aguilar-Ballester, José Tomás Real, Deborah Jane Burks, Sergio Martínez-Hervás, Herminia González-Navarro

**Affiliations:** 1 Health Research Institute Clinic Hospital of Valencia-INCLIVA, 46010 Valencia, Spain; altacor@doctor.upv.es (A.T.-C.); m.angela.vinue@uv.es (Á.V.); anhecer@alumni.uv.es (A.H.-C.); abama4@alumni.uv.es (M.A.-B.); jose.t.real@uv.es (J.T.R.); sergio.martinez@uv.es (S.M.-H.); 2 Endocrinology and Nutrition Service, Clinic Hospital of Valencia, 46010 Valencia, Spain; 3 Department of Medicine, University of Valencia, 46010 Valencia, Spain; 4 CIBERDEM (Diabetes and Associated Metabolic Diseases), 28029 Madrid, Spain; dburks@cipf.es; 5 CIPF Principe Felipe Research Center, 46012 Valencia, Spain

**Keywords:** type 2 diabetes, SGLT2i, glucose metabolism, insulin resistance, atherosclerosis

## Abstract

Type 2 diabetes mellitus (T2DM) increases morbimortality in humans via enhanced susceptibility to cardiovascular disease (CVD). Sodium-glucose co-transporter 2 inhibitors (SGLT2i) are drugs designed for T2DM treatment to diminish hyperglycaemia by reducing up to 90% of renal tube glucose reabsorption. Clinical studies also suggest a beneficial action of SGLT2i in heart failure and CVD independent of its hypoglycaemiant effect. In the present study, we explored the effect of SGLT2i dapagliflozin (DAPA) in the metabolism and atherosclerosis in *Apoe−/−Irs2+/−* mice, which display accelerated atherosclerosis induced by insulin resistance. DAPA treatment of *Apoe−/−Irs2+/−* mice, which were fed a high-fat, high-cholesterol diet, failed to modify body weight, plasma glucose or lipid. Carbohydrate metabolism characterisation showed no effect of DAPA in the glucose tolerance test (GTT) despite augmented insulin levels during the test. In fact, decreased C-peptide levels in DAPA-treated mice during the GTT suggested impaired insulin release. Consistent with this, DAPA treatment of *Apoe−/−Irs2+/−* isolated islets displayed lower glucose-stimulated insulin secretion compared with vehicle-treated islets. Moreover, insulin-signalling experiments showed decreased pAKT activation in DAPA-treated adipose tissue indicating impaired insulin signalling in this tissue. No changes were seen in lesion size, vulnerability or content of macrophages, vascular smooth muscle cells, T cells or collagen. DAPA did not affect circulating inflammatory cells or cytokine levels. Hence, this study indicates that DAPA does not protect against atherosclerosis in insulin-resistant mice in hypercholesterolemic conditions.

## 1. Introduction

Type 2 diabetes mellitus (T2DM) produces a high morbidity and mortality rate worldwide, mostly by increasing the risk of cardiovascular disease (CVD) [1,2]. CVD is caused by complications of atherosclerosis, a chronic inflammatory disease. Atheroma lesions stem from dysfunction in the endothelial layer that facilitates the accumulation of lipoproteins and immune cells in the subendothelial space of vascular vessel walls. These incipient atheroma plaques can progress to different-stage lesions, a process that is modulated by the adaptive and innate immune system. The later stages of atherosclerosis are characterised by an unbalanced interplay of immune cells that leads to an unresolved inflammatory process. Excessive inflammation and cellular death events generate clinically critic unstable plaques prone to rupture and can precipitate acute thromboembolic events [3].

Insulin resistance (IR), a primary characteristic of T2DM that may develop up to ten years before disease onset, triggers a series of mechanisms in several vascular cell types that accelerate unstable plaque formation [2]. Consequently, treatments with drugs that restore cellular and tissular insulin sensitivity could potentially reduce CVD complications in T2DM [4]. In support of this, clinical trials designed to examine the safety of newly designed T2DM drugs on heart failure (HF) and CVD have underlined the pleiotropic actions of several anti-diabetic agents [4,5]. Specifically, various investigations have shown that sodium–glucose co-transporter 2 inhibitors (SGLT2i) have beneficial mechanisms against HF and CVD [6]. In this sense, peritoneal macrophages from diabetic *Apoe−/−* and *db*/*db* mice treated with SGLT2i displayed lower foam cell formation through reduced expression of lectin-like ox-LDL receptor-1 (Lox-1), acyl-coenzyme A:cholesterol acyltransferase 1 (ACAT1) and ATP-binding cassette transporter A1 (ABCA1) [7]. SGLT2i alleviate endothelial and vascular smooth muscle cell (VSMC) dysfunction [8]. In diabetic *Apoe−/−* mice, inflammatory mediators VCAM, Mcp1 and NFκB were reduced by dapagliflozin treatment [9], empagliflozin lowered TNFα and IL6 levels [10], and canagliflozin decreased expression of VCAM and Mcp1 [11]. On the other hand, cardiomyofibroblasts from *ob*/*ob* mice treated with DAPA showed decreased Nlrp3 inflammasome and inflammatory mediators by an AMPK-dependent mechanism independent of the glucose-lowering effect through SGLT2 [12].

Under normal conditions, apical SGLT2, located in the epithelial cells of the proximal renal tubes, reabsorbs glucose and sodium which enter into the blood through basal GLUT2 transporter [13,14]. Hence, selective inhibition of SGLT2 improves T2DM by reducing glucose and sodium reabsorption by up to 90%. The main consequence of this process is that the glucose and sodium are excreted into the urine, with unique hypoglycaemic and natriuretic effects [6,14]. Nonetheless, human clinical trials have demonstrated that SGLT2i reduces body weight, blood pressure, arterial stiffness, visceral adiposity and albuminuria independently by its glucose-lowering effect [5,6]. Treatment of T2DM patients with empagliflozin added to standard drug treatment diminished incidence of CV outcomes and deaths in the EMPA-REG study [15]. Similarly, the CANVAS clinical trial, which analysed T2DM patients with chronic kidney disease treated with canagliflozin, reported a significant decrease in CVD events and kidney failure [16,17]. In line with these clinical trials, in the DECLARE–TIMI 58 trial, dapagliflozin treatment reduced the rate of hospitalisation for heart failure and of death by CVD [18]. These studies point towards safe use of SGLT2i to prevent HF, not only in T2DM but also in subjects without T2DM that have shown positive results [5,19].

In the present study, we investigated the potential benefit of a selective SGLT2i, dapagliflozin (DAPA), in atherosclerosis in *Apoe−/−Irs2+/−* mice. This is a mouse model that, under atherogenic dietary conditions, displays IR and accelerated atherosclerosis but not hyperglycaemia or hypertension, hence a highly suitable model to evaluate the impact of the drug on atherosclerosis independently from its unique hypoglycaemic and natriuretic effects.

## 2. Results

### 2.1. Metabolic Characterisation of Apoe−/−Irs2+/− Mice Treated with DAPA or Vehicle

**Figure 1:**
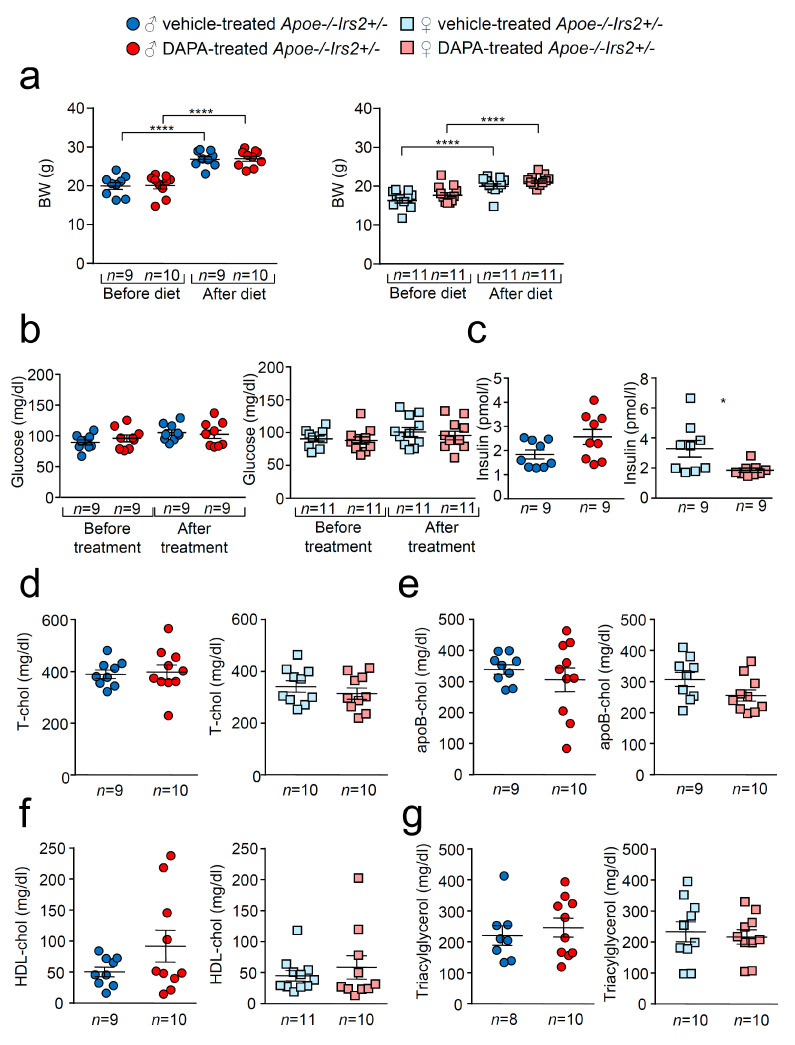
Effect of DAPA treatment in *Apoe−/−Irs2+/−* mice. (**a**) Body weight (BW) before and after atherogenic diet in male (left panel) and female (right panel) vehicle-treated and DAPA-treated *Apoe−/−Irs2+/−* mice. (**b**) Fasting plasma glucose levels in male (left panel) and female (right panel) vehicle-treated and DAPA-treated *Apoe−/−Irs2+/−* mice. (**c**) Fasting insulin levels in male (left panel) and female (right panel) vehicle- and DAPA-treated mice after treatment. (**d**) Total-cholesterol, (**e**) apoB-cholesterol, (**f**) HDL-cholesterol and (**g**) triacylglycerol levels in male (left panel) and female (right panel) DAPA- and vehicle-treated *Apoe−/−Irs2+/−* mice. Data are represented as individual points with mean ± sem. The statistical analysis for normal distribution was D’Agostino–Pearson and for differences was two-way ANOVA followed by Bonferroni’s post-hoc test (**a**,**b**), Student’s *t*-test (**c**,**f**, left panel), (**d**,**e**) and (**g**, right panel), and Mann–Whitney U test (**c**,**f**, right panel) and (**g**, left panel). **** *p* < 0.0001. * *p* < 0.05.

As expected, there was a significant increase in body weight (BW) in vehicle- and dapagliflozin(DAPA)-treated *Apoe−/−Irs2+/−* mice after receiving the atherogenic diet (Figure 1a) both in females and males. No differences were observed in BW between DAPA- and vehicle-treated mice. Similarly, fasting glucose levels remained unchanged between vehicle- and DAPA-treated male and female *Apoe−/−Irs2+/−* mice (Figure 1b). Fasting insulin levels were diminished in female DAPA-treated *Apoe−/−Irs2+/−* mice (Figure 1c, right panel) compared with vehicle-treated female controls. No changes were observed in male mice (Figure 1c, left panel). DAPA-treatment of *Apoe−/−Irs2+/−* mice did not alter fasting plasma levels of total-, apoB- and HDL-cholesterol or triacylglycerol (Figure 1d–g). Hepatic analysis also revealed no effect of DAPA-treatment on triacylglycerol content in *Apoe−/−Irs2+/−* mice (Figure S1a,b, see section on supplementary data given at the end of this article) and absence of hepatic lipid droplets (Figure S1c).

**Figure 2:**
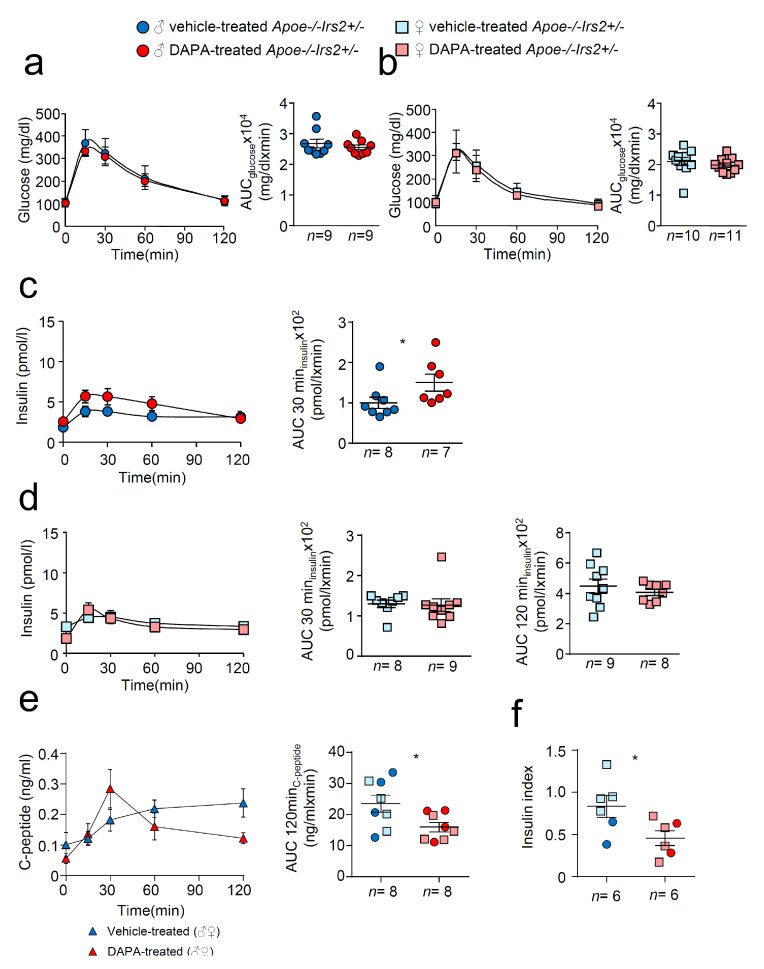
Metabolic characterisation of vehicle-treated and DAPA-treated *Apoe−/−Irs2+/−* mice. Glucose levels during the glucose tolerance test (GTT) and the area under the curve (AUC_glucose_) generated from the glucose curve during the test for male (**a**) and female (**b**) vehicle- and DAPA-treated mice. Plasmatic insulin levels stimulated by the glucose infusion during the GTT and the AUC_insulin_ calculated at 30 and 120 min of the test for (**c**) male and (**d**) female vehicle- and DAPA-treated mice. (**e**) C-peptide levels during the insulin tolerance test (ITT) and the corresponding AUC_c-peptide_ in 4-hour-fasted mice. (**f**) Insulin secretion index obtained from the glucose-stimulated insulin secretion assay in isolated islets from *Apoe−/−Irs2+/−* mice treated with DAPA or vehicle. The statistical analysis for normal distribution was D’Agostino–Pearson (**a**–**f**) and Kolmogorov–Smirnov test (**f**) and for differences Student’s *t*-test (**d**, right panel) and (**e**,**f**) and Mann–Whitney U test (**a**–**d**, left panel) (**a**–**c**) and (**d**, left panel). * *p* < 0.05.

Next, we performed a more detailed characterisation of carbohydrate metabolism. Glucose tolerance measured as the area under the curve (AUC_glucose_) parameter of the GTT was similar in both vehicle and DAPA-treated *Apoe−/−Irs2+/−* mice in both sexes (Figure 2a,b, right panels). The insulin release during the GTT, determined as AUC_insulin_ at 120 min, was also indistinguishable between vehicle- and DAPA-treated *Apoe−/−Irs2+/−* male and female mice (Figure 2c,d, right panels). Insulin release at 30 min, AUC_insulin_ 30 min (Figure 2c, middle panel) was higher in DAPA-treated male mice compared with vehicle-treated *Apoe−/−Irs2+/−* male controls. Moreover, C- peptide levels were reduced in DAPA-treated *Apoe−/−Irs2+/−* mice (Figure 2e, left panel) measured as AUC_c-peptide_ at 120 min (Figure 2e right panel) indicating that the insulin increase was due to impaired clearance rather than higher pancreatic secretion. Consistent with this, glucose stimulated insulin secretion assay, in isolated islets from *Apoe−/−Irs2+/−* mice, demonstrated diminished insulin secretion index in DAPA-treated islets (Figure 2f) compared with vehicle-treated islet controls. Thus, these results suggest that DAPA impairs insulin action in sensitive tissues and insulin secretion by islets.

**Figure 3:**
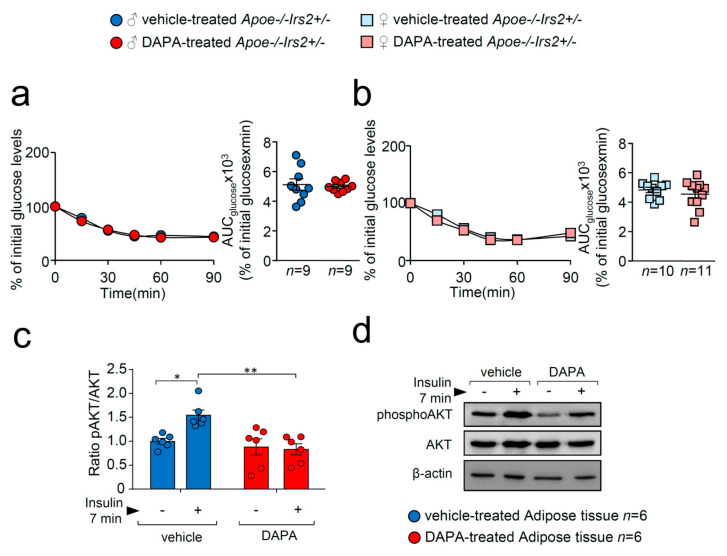
DAPA treatment effect on insulin sensitivity and signalling pathway. Glucose levels during the ITT and the corresponding AUC_glucose_ in 4-hour-fasted (**a**) male and (**b**) female *Apoe−/−Irs2+/−* mice. (**c**) Quantification of phosphorylated(activated)-AKT1/2 (pAKT1/2)/AKT1/2 ratios in insulin-stimulated adipose tissue explants pretreated with vehicle or DAPA 1 µM. β-actin is shown as a sample loading control. Phosphorylated forms were normalised to total protein levels and were relativised to the unstimulated sample. (**d**) Representative blots are depicted on the right of the quantification. Statistical analysis tests for normal distribution were the D’Agostino–Pearson omnibus (**a**,**b**) and Kolmogorov–Smirnov test (**c**) and for differences, the Student’s *t*-test (**a**,**b**) and two-way ANOVA followed by Tukey’s multiple comparison test (**c**). * *p* < 0.05; ** *p* < 0.01.

To better analyse insulin action in DAPA-treated *Apoe−/−Irs2+/−* mice, insulin sensitivity and signalling were explored. Insulin sensitivity assessed by the insulin tolerance test (ITT) and measured by the AUC_glucose_ revealed no effect of DAPA in *Apoe−/−Irs2+/−* mice neither in males (Figure 3a) or in females (Figure 3b). However, analysis of the insulin-signalling pathway activation in vitro showed decreased pAKT/AKT protein ratio levels in DAPA-treated adipose tissue explants (Figure 3c).

These results indicate that DAPA does not have a beneficial effect on carbohydrate metabolism in *Apoe−/−Irs2+/−* mice and might even exert detrimental effects on insulin signalling and insulin secretion in certain conditions.

### 2.2. DAPA Treatment in Atherogenic Diet-Fed Apoe−/−Irs2+/− Mice Does Not Affect Atherosclerosis Lesion Size or Plaque Stability

**Figure 4:**
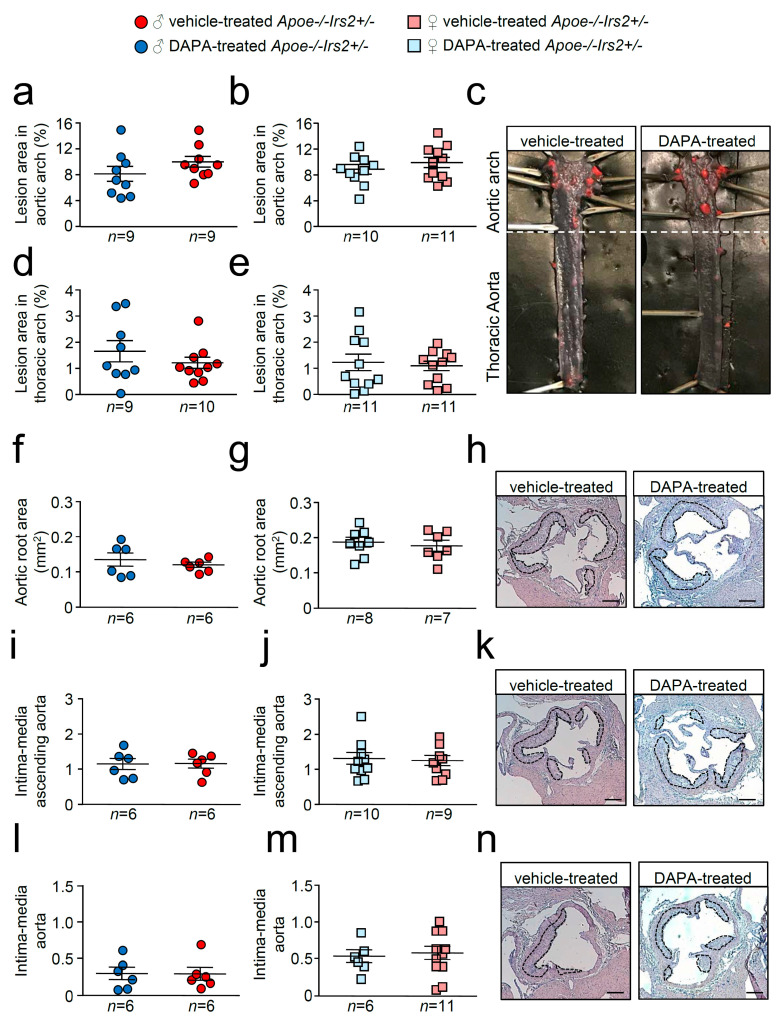
Atherosclerotic lesion size analysis in vehicle- and DAPA-treated *Apoe−/−Irs2+/−* mice. Atherosclerosis analysis in (**a**,**b**) the aortic arch and (**d**,**e**) the thoracic aorta of whole-mounted oil-red-O-stained aortas from both vehicle- and DAPA-treated male and female mice. Lesion size determinations in cross-sections of (**f**,**g**) the aortic root, in (**i**,**j**) the ascending aorta, and in (**l**,**m**) the aorta displayed as absolute area (**f**,**g**) and as an intima-media ratio (**i**,**j**,**l**,**m**). (**c**) Representative photographs of whole-mounted aortas. Magnification 0.17. Representative images of haematoxylin/eosin-stained cross-sections of the aortic root (**h**), ascending aorta (**k**) and aorta (**n**). The limits of the lesion are indicated by the dashed lines. Scale bar: 200 µm. The statistical analysis for normality was D’Agostino–Pearson omnibus test and for differences was the Student’s *t*-test (**a**,**b**,**d**,**j**) and the Mann–Whitney U test (**e**–**g**,**i**,**l**,**m**).

*En face* atheroma lesion size determination in whole–mounted aortas showed no effect of DAPA-treatment in aortic arches or thoracic aortas of *Apoe−/−Irs2+/−* mice compared with vehicle-treated controls regardless of sex (Figure 4a–e). Atherosclerosis development determinations in cross-sections revealed similar sizes in male and female DAPA- and vehicle-treated *Apoe−/−Irs2+/−* mice in the three regions analysed, aortic root (Figure 4f–h), ascending aorta (Figure 4i–k), and aorta (Figure 4l–n).

**Figure 5:**
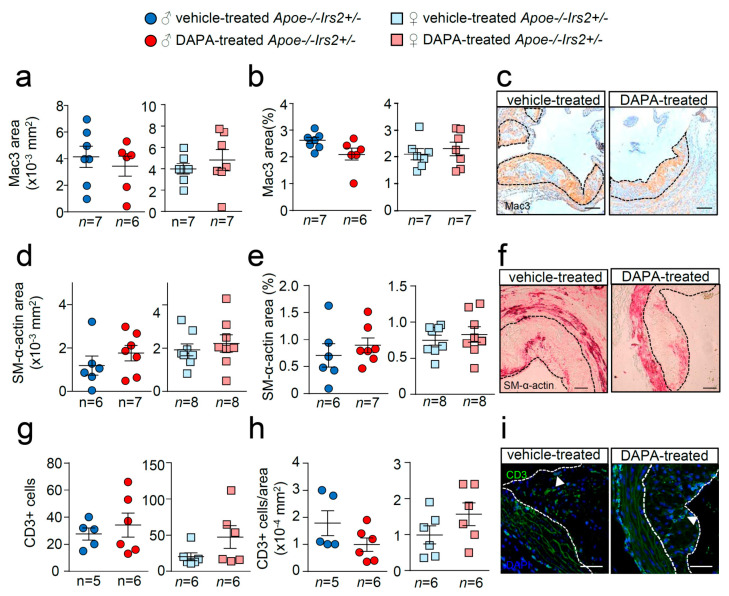
Effect of DAPA in atheroma plaque characteristics of *Apoe−/−Irs2+/−* mice. (**a**,**b**) Macrophage and (**d**,**e**) vascular smooth muscle cell (VSMC) content in aortic cross-sections from vehicle- and DAPA-treated male (left panels) and female mice (right panels), determined as Mac3+ and SM-α-actin+ areas measured as absolute area (in mm^2^) (**a**,**d**) and as percentage of the positive-stained area relative to lesion area (**b**,**e**). Scale bar: 100 µm. Number of T lymphocytes in lesions of aortic cross-sections detected as CD3+ cells and depicted as (**g**) absolute cell number and as (**h**) cell number relative to lesion area (in mm^2^) in both sexes from vehicle- and DAPA-treated mice. Scale bar: 50 µm. Photomicrographs next to the quantifications (**c**,**f**,**i**) show representative images of the immunostainings. White arrows in (**i**) point to CD3+ cells. The limits of the lesion are indicated by the dashed lines. The statistical analysis for normal distribution was the D’Agostino–Pearson omnibus test (**d**,**e**) and Kormokov–Smirnov was performed using Student’s *t*-test (**d**,**e**. right panels) and Mann–Whitney U test (**a**,**b**,**g**,**h**) and (**d**,**e**, left panels).

Further analysis of the atheroma cellular composition and parameters of plaque stability was next performed. Macrophage, vascular smooth muscle cells (VSMCs), and T lymphocyte absolute and relative contents were undistinguishable between male and female DAPA- and vehicle-treated *Apoe−/−Irs2+/−* mice (Figure 5a–i).

**Figure 6:**
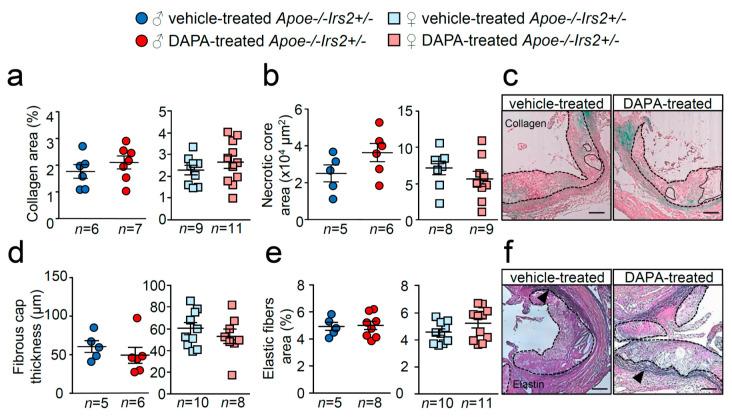
Effect of DAPA in atheroma plaque characteristics of *Apoe−/−Irs2+/−* mice. (**a**) Collagen content (in percentage), (**b**) necrotic core area (in µm^2^) and (**d**) fibrous cap thickness (in µm) determined in Masson trichrome stained lesions from vehicle- and DAPA-treated male (left panels) and female (right panels) mice. (**e**) Elastin fibres’ area assessed in Verhoeff-Van Gieson cross-sections and displayed as percentage of area relative to lesion area in male and female mice. Representative images of Masson trichrome (**c**,**f**) Verhoeff-Van Gieson stainings. Black arrows in (**f**) point to elastic fibre’s breaks in the media. The dashed lines delineate the limits of the lesion and solid lines delineate the limits of representative necrotic cores. Scale bar: 100 µm. The statistical analysis for normal distribution was Kolmogorov–Smirnov test and for differences was the Student’s *t*-test (**a**,**b**,**d**,**e**, right panels) and Mann–Whitney U test (**a**,**b**,**d**,**e**, left panels).

Similarly, examination of collagen content, necrotic core area, fibrous cap thickness and elastic fibre’s area failed to show differences between treatments (Figure 6a–f). Thus, DAPA treatment in *Apoe−/−Irs2+/−* mice on a 2-month atherogenic diet does not influence atherosclerosis development or plaque stability parameters.

### 2.3. DAPA Treatment Effect in Inflammation in Apoe−/−Irs2+/− Mice Fed an Atherogenic Diet

Some investigations have suggested a role for gliflozins in inflammation and in heart failure through monocyte/macrophage polarisation and, in light of this, inflammatory parameters were studied.

**Figure 7:**
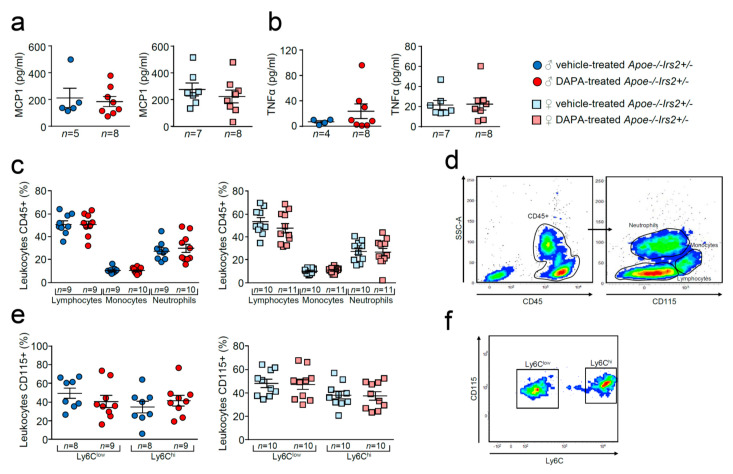
Effect of dapagliflozin in inflammatory mediators and cells in *Apoe−/−Irs2+/−* mice. Circulating plasma levels of (**a**) MCP1 and (**b**) TNFα proinflammatory cytokines. (**c**) Circulating levels (in percentage) of lymphocytes, monocytes and neutrophils in the CD45+ population, identified by morphology and CD115 monocyte marker. (**d**) Representative cytometry plots of the gating strategy used for the flow cytometry analysis in the different leukocyte populations. (**e**) Circulating levels of monocyte subpopulations (in percentage) identified as CD45+CD115+Ly6C^low^ pro-resolving and as CD45+CD115+Ly6C^hi^ invasive monocyte subsets in male and female mouse blood. (**f**) Representative cytometry plots of the gating strategy used for monocyte subsets in blood samples. The statistical analyses for normal distribution were Kolmogorov–Smirnov test (**a**,**b**) and D’Agostino–Pearson test (**c**,**e**) and for differences were Mann–Whitney U test (**a**,**b**) and Student’s *t*-test (**c**,**e**).

Compared with vehicle-treated *Apoe−/−Irs2+/−* mice, DAPA-treated mice did not show differences in the circulating plasma levels of MCP1 or TNFα (Figure 7a,b). Analysis of circulating leukocytes showed similar percentages of lymphocytes, monocytes, and neutrophils between DAPA- and vehicle-treated *Apoe−/−Irs2+/−* male and female mice (Figure 7c,d). Monocyte subpopulations analysis did not detect changes in the proinvasive and proinflammatory Ly6C^hi^ subsets or in the pro-resolving Ly6C^low^ monocyte subpopulations in DAPA-treated *Apoe−/−Irs2+/−* mice compared with vehicle-treated controls (Figure 7e,f).

Likewise, T CD3+ CD4+ and CD8+ lymphocytes populations (Figure S2a,d,g) and their activated subpopulations CD3+CD69+, CD4+CD69+ and CD8+CD69+ (Figure S2b,e,h) remained unchanged. Circulating CD4+CD25+Foxp3+ Treg cell analysis showed also similar levels in DAPA- and vehicle-treated *Apoe−/−Irs2+/−* mice (Figure S2j).

### 2.4. Effect of DAPA in Apoe−/−Irs2+/− Mouse Macrophages

A recent investigation has shown that DAPA attenuates myocardial infarction through macrophage phenotype modulation [20], therefore, the effects of DAPA and DAPA conditioned media (DAPACM) and their respective vehicle controls were evaluated in in vitro *Apoe−/−Irs2+/−* murine-derived macrophages.

**Figure 8:**
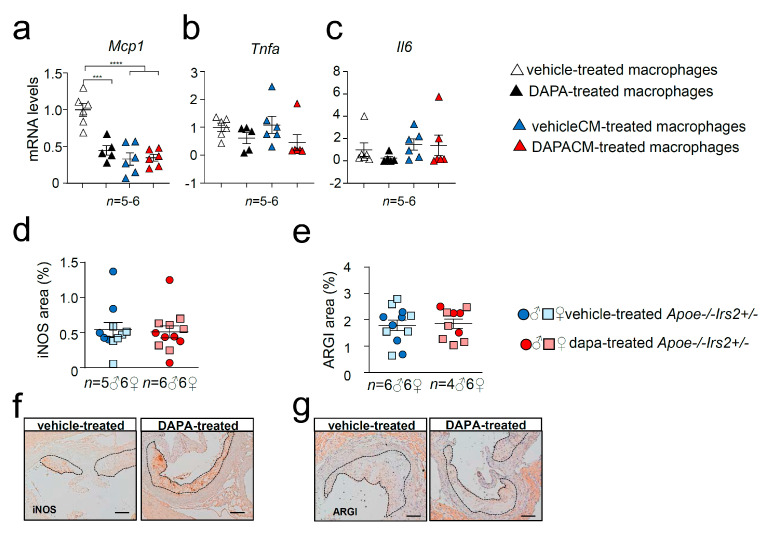
Effect of DAPA in *Apoe−/−Irs2+/−* mouse macrophage phenotype. mRNA expression of proinflammatory (**a**) *Mcp1*, (**b**) *Tnfa* and (**c**) *Il6* cytokines in *Apoe−/−Irs2+/−* macrophages treated with vehicle (vehicle-treated), 1µM of DAPA (DAPA-treated) or with conditioned media containing 10% of plasma from vehicle-treated mice (vehicleCM) or with conditioned media containing 10% of plasma from DAPA-treated mice (DAPACM). (**d**) iNOS and (**e**) ARGI lesional content in aortic cross-sections from vehicle- and DAPA-treated male and female mice, determined as percentage of positive stained area relative to lesion area. Representative images of the (**f**) iNOS, and (**g**) ARGI, stainings used for the quantifications are shown. Scale bar: 100 µm. The lesion is limited by dashed lines. The statistical analysis for normal distribution was Kolmogorov–Smirnov test and for differences was one-way ANOVA followed by Tukey’s multiple comparison test (panel **a**), Kruskal–Wallis followed by Dunn’s multiple comparison test (panels **b**,**c**) and Mann–Whitney U test (panels **d**,**e**). *** *p* < 0.001; **** *p* < 0.0001.

Cytokine expression analysis revealed diminished mRNA levels of *Mcp1* in DAPA-treated (Figure 8a) macrophages compared with vehicle-treated cells without changes in *Tnfa* (Figure 8b) or *Il6* (Figure 8c) expression. Consistent with the in vivo data, cytokine gene expression in macrophages was not altered by the treatment with media containing DAPA-treated mouse plasma, DAPACM, compared with that in macrophages treated with vehicleCM derived from vehicle-treated mice (Figure 8a–c). Analysis of surface macrophage markers showed no differences in the activation marker *Cd11c* among treatments (Figure S3a). DAPA-treated macrophages displayed diminished expression of the *Cd206* macrophage marker compared with controls (Figure S3b) but remained unchanged between DAPACM and vehicleCM treatments (Figure S3b). Consistent with the results in macrophages treated with conditioned media, the content of macrophage markers iNOS and ARGI in atheroma lesions was similar between DAPA- and vehicle-treated *Apoe−/−Irs2+/−* mice (Figure 8d,e).

## 3. Discussion

T2DM is a major risk factor for developing atherosclerosis, the main cause of CVD. Human clinical trials have shown that anti-diabetic drug SGLT2i protects against CVD and HF in T2DM beyond its glucose-lowering effects. Several anti-diabetic agents control atherosclerosis development by restoring the homeostasis altered by IR in atheroma lesional cells, a key feature of T2DM. Therefore, in the present study we evaluated the potential of SGLT2i DAPA in restraining atherosclerosis in IR conditions. To this end, we investigated the effect of DAPA on *Apoe−/−Irs2+/−* mice, which, under cholesterol-enriched atherogenic diet conditions, display accelerated atherosclerosis, IR and hypercholesterolemia. Treatment of *Apoe−/−Irs2+/−* mice with DAPA did not result in changes in BW or fasting plasma glucose compared with vehicle-treated controls. Fasting insulin was increased in DAPA-treated females but not in males. Similarly, no differences in the levels of cholesterol (total, apoB- and HDL-cholesterol) or triacylglycerol were observed between vehicle- and DAPA-treated mice. Carbohydrate metabolism characterisation revealed no effect of DAPA on glucose tolerance or insulin sensitivity tests. Notwithstanding this, analysis of insulin release during the GTT demonstrated increased insulin levels but lower C-peptide release in DAPA-treated *Apoe−/−Irs2+/−* mice indicating abnormal insulin clearance. Further analysis showed impaired insulin secretion of isolated pancreatic islets treated with DAPA. In addition, impaired insulin signalling in adipose tissue explants pretreated with DAPA was observed suggesting a detrimental effect on insulin action in this tissue. Atherosclerosis lesion determinations in whole-mounted aorta and heart cross-sections did not show differences between vehicle and DAPA-treated mice. No effect of DAPA was observed in cellular and collagen contents or in plaque vulnerability parameters within atheroma lesions. Likewise, DAPA did not affect circulating immune system cells or plasmatic cytokine levels in vivo, although in macrophages resulted in a decrease in *Mcp1* and *Cd206* expression compared to vehicle-treated macrophages. Altogether this indicates that DAPA treatment in vivo does not protect against atherosclerosis in *Apoe−/−Irs2+/−* mice and it seems to interfere with islet insulin secretion and adipose tissue insulin-signalling. These data suggest that DAPA might not be effective against atherosclerotic complications associated with IR or prediabetic states.

SGLT2i has been widely demonstrated to exert protective effects in metabolism by reducing plasmatic hyperglycaemia and increasing glucosuria and natriuresis. Weight loss due to urinary loss of calories, improved renal function and blood pressure are direct consequences of SGLT2i [21,22]. Moreover, in T2DM subjects, empagliflozin treatment improved insulin β cell function and insulin sensitivity [23] while DAPA only ameliorated muscle insulin sensitivity [24]. In our study, with a mouse model exhibiting IR but no other T2DM features, which also had hyperlipidaemia due to a high-fat, high-cholesterol diet, DAPA treatment during the glucose challenge did not change glucose levels, despite increased insulin plasmatic concentration. Blood insulin levels are determined by pancreatic islet secretion but also by its plasmatic clearance. In fact, the observed decreased C-peptide release suggested impaired insulin secretion from pancreatic islets. Consistent with the latter, isolated pancreatic islets from *Apoe−/−Irs2+/−* mice treated with DAPA exhibited diminished insulin secretion index. Hence, the defective blood glucose lowering effect of the circulating insulin in DAPA-treated mice indicated otherwise defective clearance. Of note, defective insulin clearance and degradation are observed in subjects with T2DM frequently due to abnormal insulin–insulin receptor (INSR) internalisation and recycling in insulin-sensitive tissues, including liver, skeletal muscle, adipose tissue and kidney [25,26]. Consistent with defective INSR downstream signalling, experiments in adipose tissue demonstrated impaired insulin-pathway activation, shown by a reduction in phosphoAKT protein levels. Our results suggest that DAPA might affect metabolism by interfering with insulin-signalling in adipose tissue. Notwithstanding, given that the major contributors to insulin clearance are hepatic and skeletal muscle, further research must be conducted to demonstrate the hypothesis. Altogether, our study suggests that DAPA treatment in IR mice in hyperlipidaemic conditions might have limited effects.

In addition, DAPA did not alter atherosclerosis or inflammatory parameters in our mouse model. Preclinical investigations have shown that DAPA does not act on atherosclerosis in *Apoe−/−* mice, but significantly diminished lesion size in hyperglycaemic conditions without IR. Thus DAPA decreased atheroma size in streptozotozin (STZ)-induced hyperglycaemic and diabetic *Apoe−/−* mice through reduction in hyperglycaemia-induced foam cell formation [7]. On the other hand, empagliflozin treatment decreased atheroma plaques of *Apoe−/−* mice fed a carbohydrate- (43%) and fat- (41%) rich Western diet [10]. Although the authors suggested that IR improvement was the precipitant of atherosclerosis reduction, empagliflozin treatment also decreased carbohydrate-induced hyperglycaemia, which could help mitigate atherosclerosis development. In agreement with this, DAPA reduced atherosclerosis in STZ-induced diabetic *Ldlr−/−* [27] and *Apoe−/−* [28] mice. Consistent with these studies, empagliflozin [29] and canagliflozin [30] ameliorated endothelial dysfunction and atheroma lesion development in STZ-induced diabetic *Apoe−/−* mice. Empagliflozin also facilitated regression of atherosclerosis in STZ-induced hyperglycaemic mice whose atherosclerosis was induced by *Ldlr−* and *Srb1−* antisense oligonucleotide injection [31]. Altogether, these studies cast doubt on the beneficial actions of SGLTi in the absence of hyperglycaemia and suggest that anti-atherogenic effects are exerted by decreasing glucose levels independently of insulin action.

Among SGLT2i mechanisms [32], polarisation towards a non-invasive and pro-resolving phenotype of macrophages via increased signal transducer and activator of transcription 3 (STAT3) activity-dependent mechanism has been reported [20]. Notably, we have previously demonstrated that glucagon-like peptide 1 receptor (GLP1R) agonists decrease plaque size and vulnerability in *Apoe−/−Irs2+/−* mice by inducing a protective circulating immune cell phenotype, and by inducing polarisation of lesional macrophage to an anti-inflammatory phenotype [33]. Studies in the present investigation showed that in vitro treatment of macrophages with DAPA reduced expression of proinflammatory *Mcp1* cytokine and macrophage marker *Cd206*. However, here DAPA did not show an in vivo impact on either circulating immune cells or lesional macrophage content or markers (iNOS and ARGI). Therefore, our results show that although DAPA might have potential beneficial effects on macrophages in vitro, these are irrelevant for in vivo macrophage phenotype modulation and for atherosclerosis development in our experimental setting, characterised by the absence of hyperglycaemia.

In summary, our study indicates that DAPA treatment fails to alter glucose levels or atherosclerosis development in *Apoe−/−Irs2+/−* mice, which develop hypercholesterolemia and IR but not hyperglycaemia. Moreover, DAPA reduced glucose-stimulated insulin secretion from islets, insulin clearance in vivo and insulin-signalling in adipose tissue. The present investigation suggests that DAPA might not exert beneficial effects in atheroma vascular disease in IR and hypercholesterolemic conditions.

## 4. Materials and Methods

### 4.1. Mice, Diets and Drug Treatments

*Apoe−/−* and *Irs2+/−* mice were crossed to generate *Apoe−/−Irs2+/−* mice (C57BL/6J background, *n* = 21 male and *n* = 22 female). Mice had free access to water, were under temperature and humidity-controlled conditions (22 ± 2 °C, 55 ± 10%) and with 12h light–dark cycles (8:00–20:00 h) in a conventional animal facility. Mice were maintained on a regular chow diet (Teklad Global Rodent Diets 6.5% fat; Tekland, Envigo, Barcelona, Spain) and at 8 weeks of age were placed on an atherogenic diet (10.8% fat, 0.75% cholesterol, S4892-E010, Ssniff, Soest, Germany) which is a Paigen-based diet with modifications as described before [34] for 8 weeks. For the last 6 weeks, mice were randomly assigned to receive daily treatment with dapagliflozin (*n* = 11 female *n* = 11 male; p.o. 3 mg/kg/day in 120 µL) or with vehicle [35] (carboxymethyl cellulose) (*n* = 10 female *n* = 11 male). Dose, timing of treatment, route of administration and drug concentration for delivery was based on previous investigations [33,35]. All animal procedures were approved by the Animal Ethics Committee of INCLIVA and University of Valencia (5/25/2017, procedure A1484231073016, administration approval 2017/VSC/PEA/00081) and complied with the 2010/63/EU European Parliament directive.

### 4.2. Plasmatic Biochemical Determinations and Metabolic Assays

Plasma was obtained from whole blood with ethylenediaminetetra-acetic acid (EDTA) of overnight-fasted mice. Analysis included triacylglycerol, total cholesterol, apoB-cholesterol, HDL-cholesterol (Wako Diagnostics, Mountain View, CA, USA) as previously described [36]. Glucose tolerance tests (GTTs) (2 g/Kg of body weight, BW, of glucose) and insulin tolerance tests (ITTs) (0.5 U/Kg of BW Humulina, Lilly, Alcobendas, Spain) were performed in overnight- and 4-h-fasted mice, respectively. Glucose levels were measured at different time points using a glucometer (Ascensia Elite, Bayer, Leverkusen, Germany). Fasting basal insulin and insulin-release and C-peptide levels during the GTT were measured using an anti-mouse insulin and C-peptide ELISA kit (CrystalChem, Zaandam, The Netherlands) as reported [36].

### 4.3. Pancreatic Islet Isolation and Glucose-Stimulated Insulin Secretion Assay

For islet isolation, *Apoe−/−Irs2+/−* mice were infused with Krebs buffer (127 mM NaCl, 5 mM KCl, 3 mM CaCl_2_, 1.5 mM MgCl_2_, 24 mM NaHCO_3_, 6 mM Hepes, 2 mg/mL glucose, 0.1% albumin, equilibrated with 5% CO_2_ in O_2_) and the pancreas was dissected and digested with collagenase-NB8 (1 mg/mL, Serva, Heidelberg, Germany) at 37 °C in a shaking water bath for 20 min. Islets were handpicked under stereoscope as described [37]. Insulin secretion was evaluated by a glucose-stimulated insulin secretion assay at low (2.8 mmol/L) and high (16.7 mmol/L) glucose concentrations in KRBH buffer (140 mM NaCl, 2.5 mM KCl, 2.5 mM CaCl_2_, 1 mM MgCl_2_, 20 mM Hepes, 2 mg/mL glucose, 0.1% albumin). Four assays (5 islets each) were performed per condition and insulin concentrations were measured by ELISA. The glucose stimulated insulin secretion index was calculated as the ratio between high glucose-stimulated insulin release and the low glucose-stimulated insulin secretion normalised each by the insulin content of the islets.

### 4.4. Liver Triacylglycerol Content and Histochemical Staining

Triacylglycerol hepatic content was determined by tissue digestion and saponification in ethanolic potassium hydroxide, followed by enzymatic measurement of glycerol content (Free Glycerol Reagent, Sigma, St. Louis, MO, USA). For examination of lipid droplets in hepatic tissue, paraffin-embedded tissue sections were stained with haematoxylin–eosin and analysed as described [36].

### 4.5. Insulin Signalling Experiments in Adipose Tissue and Protein Analysis by Western Blot

Epididymal visceral fat pads from *Irs2+/−* mice were minced into 2–3 mm^3^ and incubated 24 h at 100 mg/mL in 6 well-plates with DMEM-P/S/A-10%FBS media in a humidified 5% CO_2_ atmosphere. Explants were washed with PBS1X twice and incubated with DMEM-P/S/A-0.1%FBS media for 24 h in the presence of DAPA 1 µM or media with vehicle as control adipose tissue. Explants were then stimulated 7 min with insulin (200 nM, Sigma, St. Louis, MO, USA) or vehicle and then snap-frozen in liquid N_2_ for insulin-signalling pathway analysis by Western blot as described [38]. Briefly, protein extracts were obtained by homogenising adipose tissue in the presence of ice-cold fat lysis buffer (Hepes 50 mM, pH 7.5, NaCl 150 mM, Triton 1% vol/vol, glycerol 10% vol/vol) supplemented with Complete Mini cocktail, PhosSTOP (Roche, Mannheim, Germany), beta-glycerophosphate 50 mM (Sigma), 2 mM phenylmethylsulfonyl fluoride (PMSF, Roche) and 200 µM Na_3_VO_4_ (Sigma). Protein extracts (50 µg) were prepared with Laemmli buffer (5 min 95 °C) and subjected to 12% *w/v* polyacrylamide gel electrophoresis and Western blot as described. The following primary (1/200) and secondary (1/2000) antibodies were used to detect the proteins: pAKT (4058, Cell Signalling, Beverly, MA, USA), AKT1/2 (sc1619, Santa Cruz Biotech Dallas, TX, USA) and β-actin (A5441, Sigma, St. Louis, MO, USA), anti-mouse IgG-HRP (P0447, Dako), donkey anti-goat IgG-HRP (sc2056, Santa Cruz Biotech, Dallas, TX, USA) and goat anti-rabbit IgG-HRP (P0448, Dako Agilent, Santa Clara, CA, USA). The immunocomplexes were detected with an ECL Plus detection kit (ThermoFisher, Waltham, MA, USA). All antibodies for Western blot were acquired and used after checking that validation was performed by manufacturer company.

### 4.6. Atheroma Lesion Analysis

To quantify atheroma lesion size, aortas and hearts of mice were dissected and fixed with 4% paraformaldehyde/PBS (vol/vol). Lesion extension was assessed by an *en face* analysis of whole-mounted aortas stained with oil red-O (Sigma) using morphometric analysis (Fiji, ImageJ-win64, NIH, Bethesda, MD, USA). Hearts were paraffin-embedded, sectioned for haematoxylin/eosin staining and analysed for atheroma lesion size measured as lesion area (mm^2^) in the aortic root and as the intima–media ratio in two regions of the ascending aorta [40].

### 4.7. Histopathological Characterisation of Atheroma Lesions

Collagen content, necrotic core area, and fibrous cap thickness of atheromas were determined in sections stained with Masson’s trichrome staining. Elastic fibres were analysed in aortic cross-sections stained with Verhoeff-Van Gieson staining. For plaque instability, fibrous cap thickness (in µm) and necrotic core area (measured as µm^2^) were determined. The fibrous caps were defined as regions above the necrotic cores and were later identified as non-stained acellular areas [39]. Macrophages in lesions were detected by immunohistochemistry in cross-sections treated for peroxidase inactivation (H_2_O_2_ 0.3%) and blocking with horse serum (5%) followed by incubation overnight at 4 °C with an anti-Mac-3 antibody (1/200, Santa Cruz Biotechnology, Santa Cruz, CA, USA) and biotin-conjugated goat anti-rat secondary antibody incubation 1 h at room temperature (1/300, Santa Cruz). For inducible nitric oxide synthase (iNOS) and arginase I (ARGI) analysis, aortic cross-sections underwent antigen retrieval (citrate buffer, 10 mM, pH 6.5 high pressure and temperature), peroxidase inactivation (H_2_O_2_ 3%), blocking with horse serum (5%), incubation overnight at 4 °C with primary antibodies (rabbit anti-MMP-9, 1/200 dilution, (UPSTATE-Millipore, Billerica, MA, USA), anti-iNOS antibody 1/100 dilution (Abcam, Cambridge, UK) and anti-arginase I 1/50 dilution (Sigma, St. Louis, MO, USA)), and incubation with a biotinylated anti-rabbit secondary antibody (1/500 dilution, Santa Cruz). Immunocomplexes were detected with streptavidin-HRP (ab7403, Abcam, Cambridge, UK) and then developed with DAB substrate. Counterstaining was done with haematoxylin and slides were mounted with EUKITT (Deltalab, Barcelona, Spain). Lesional VSMCs were detected using an anti-SMα-actin monoclonal alkaline phosphatase-conjugated antibody (1/20 dilution, Sigma) and Fast Red substrate (Sigma). Slides were mounted with glycerol gelatin (Sigma) [33]. All images were captured with a Leica DMD108 microscope coupled to a camera (Leica, Wetzlar, Germany). T-lymphocytes in atheromas were detected by immunofluorescence with a polyclonal anti-human CD3 antibody (1/75 dilution, Dako, Santa Clara, CA, USA) and Alexa Fluor^®^-488 anti-rabbit IgG secondary antibody (1/200 dilution, Invitrogen, Madrid, Spain). Slides were mounted with Slow-Fade Gold reagent (Invitrogen) and analysed with an inverted fluorescent microscope (DMI3000B, Leica). All antibodies used were validated using negative controls by omission of primary antibodies.

### 4.8. Enzyme-Linked ImmunoSorbent Assay (ELISA) of Cytokines

Circulating levels of cytokines were determined in isolated plasma from heparinised mouse blood (10U heparin/mL) using the specific cytokine DuoSet ELISA kits (R&D Systems, Minneapolis, MN, USA).

### 4.9. Circulating Leukocyte Analysis by Flow Cytometry

To characterise circulating leukocytes by flow cytometry, 10 µL of heparinised whole blood was incubated for 30 min at RT with CD45-FITC (BD Biosciences, Franklin Lakes, NJ, USA) to identify leukocytes. Lymphocytes and neutrophils were identified by complexity and morphology, whereas monocytes were gated based on the CD115-APC marker (Biolegend, San Diego, CA, USA). Ly6C-PerCP (BD Biosciences, San Diego, CA, USA) and CD115-APC (Biolegend, San Diego, CA, USA) were used for Ly6C^low^ and Ly6C^hi^-monocyte subsets identification. For circulating lymphocytes analysis, 10 µL of heparinised whole blood was incubated with 5 µL Brilliant Stain Buffer (BD Biosciences, San Diego, CA, USA) followed by CD4-BV421, CD8a-BV510, CD3e-APC, or CD69-PE antibodies (BD Biosciences, San Diego, CA, USA). Mouse Tregs were identified using Kit FoxP3 Staining Buffer Set and with anti-CD25-APC, anti-Foxp3-PE, and anti-CD4-VioBlue (all from Miltenyi, Bergisch Gladbach, Germany). The samples were incubated with FACS Lysing solution (BD Biosciences) for 10 min before flow cytometry analysis (FACSVerse or FACS Fortessa Flow cytometer, BD Biosciences, San Diego, CA, USA).

### 4.10. Experiments with Bone Marrow-Derived Macrophages

Murine bone marrow-derived macrophages were obtained from the femoral and tibial bone marrow of mice sacrificed by cervical dislocation. Bone marrow cells were differentiated for 5–6 days with 10% FBS/DMEM (Lonza, Basel, Switzerland) supplemented with 10% L929-cell conditioned medium (LCM: as a source of macrophage colony-stimulating factor) [40]. For the last 24 h, macrophages were treated with 0.5%FBS/DMEM medium supplemented with vehicle, 1 µm of DAPA, 10% of plasma from DAPA-treated mice (Dapagliflozin-conditioned media, DAPACM) or 10% of plasma from vehicle-treated mice (vehicle-conditioned media, vehicleCM). Cytokine gene expression was analysed by qPCR.

### 4.11. Gene Expression Analysis by Quantitative Real-Time PCR

RNA (0.5–1 μg) from murine macrophages was obtained using TRIzol Reagent (Invitrogen), which was reverse-transcribed with the Maxima First Strand cDNA Synthesis kit and amplified with Luminaris Color HiGreen High ROX qPCR Master Mix (Thermo Scientific, Waltham, MA, USA) on a thermal Cycler 7900 FastSystem. Results were analysed with the provided software (Applied Biosystems, Foster city, CA, USA). mRNA levels were normalised to cyclophilin expression and relativised to controls. The primer sequences can be found in Supplementary Data Table S1.

### 4.12. Statistical Analysis

Quantitative variables are shown as single data points and mean ± sem. All data obtained were analysed unless these data were out of range of the standard curve or if samples were lost during the experimentation. Data were analysed for normal distribution using the D’Agostino–Pearson omnibus, Shapiro–Wilk test or Kolmogorov–Smirnov test. Differences were evaluated with the Student´s *t*-test, Mann–Whitney U test, two-way ANOVA followed by Bonferroni or Tukey’s post hoc test, one-way ANOVA followed by Bonferroni or Tukey’s multiple comparison test (more than two groups), or Kruskal–Wallis followed by Dunn’s multiple comparison test (more than two groups) (GraphPadPrism Software Inc., La Jolla, CA, USA). Statistical significance was set at *p* ≤ 0.05. Male and female data were displayed separately.

## 5. Conclusions

Altogether, our study indicates that in mice with IR but without hyperglycaemia or other characteristics of T2DM, DAPA treatment does not restrain atherosclerosis, and could have detrimental effects.

## Supplementary Materials

Supplementary materials can be found at https://www.mdpi.com/1422-0067/21/23/9216/s1.

## Abbreviations

Apoe	apolipoprotein E
ARG	arginase I
AUC	area under the curve
BW	body weight
CVD	cardiovascular disease
DAPA	dapagliflozin
EDTA	ethylenediaminetetra-acetic acid
ELISA	enzyme-linked immunosorbent assay
GTT	glucose tolerance test
HF	heart failure
iNOS	inducible nitric oxide synthase
IR	insulin resistance
IRS2	insulin receptor substrate 2
ITT	insulin tolerance test
MMP9	matrix metallopeptidase 9
qPCR	quantitative polymerase chain reaction
SGLT2	sodium-glucose transporter 2
SGLT2i	sodium-glucose transporter 2 inhibitor
STAT	signal transducer and activator of transcription
STZ	streptozotocin
T2DM	type 2 diabetes mellitus
VSMC	vascular smooth muscle cells
