# Supplementary material for: Dapagliflozin Does Not Modulate Atherosclerosis in Mice with Insulin Resistance
Source: Int J Mol Sci. 2020 Dec 3;21(23):9216. doi: 10.3390/ijms21239216 (PMC7730942; doi:10.3390/ijms21239216)
Supplement: Supplementary file 1 [file ijms-21-09216-s001.pdf]

Supplementary Data

Dapagliflozin does not modulate atherosclerosis in mice with insulin resistance

Alida Taberner-Cortés[1], Ángela Vinué1, Andrea Herrero-Cervera[1], María Aguilar-Ballester[1], José Tomás Real[1,2,3] Deborah Jane Burks[3,4] , Sergio Martínez-Hervás[1, 2, 3] and Herminia González-Navarro[1,3,#]

Supplementary Tables

Table S1. Sequences of primers used for gene expression analysis by qPCR

Gene	Primer	Sequence 5′→3′
Cyclophilin	Forward	AGATGGAGAACCGACGTATCA
	Reverse	ACTGAGCGTGCTGACAAGTC
Cd11c	Forward	ATGGAGCCTCAAGACAGGAC
	Reverse	GGATCTGGGATGCTGAAATC
Cd206	Forward	GCATGGGTTTTACTGCTACTTGATT
	Reverse	CAGGAATGCTTGTTCATATCTGTCTT
Il6	Forward	CCCAACAGACCTGTCT
	Reverse	CCAGTTTGGTAGCATCC
Mcp1	Forward	CTTCCTCCACCACCATGCA
	Reverse	CCAGCCGGCAACTGTGA
Tnfa	Forward	CCCACACCGTCAGCCGATTT
	Reverse	GTCTAAGTACTTGGGCAGATTGACC

Supplemental Figures

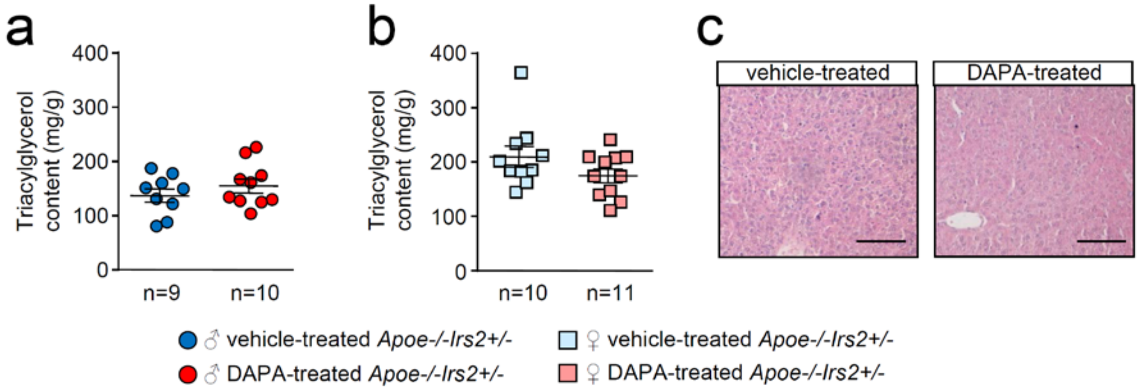

Figure S1. Hepatic triglyceride content in vehicle- and DAPA-treated *Apoe-/-Irs2+/-* mice. Hepatic triglyceride content (mg/g liver tissue) in **(a)** male and **(b)** female mice. **(c)** Representative images of hematoxylin-eosin stained cross-sections of vehicle- and DAPA-treated *Apoe-/-Irs2+/-* mice. Scale bar: 100 µm. Data is represented as individual points with mean ± sem The statistical analysis for normality was D'Agostino-Pearson omnibus test and for differences were the student's t-test (a) and Mann-Whitney U test (b).

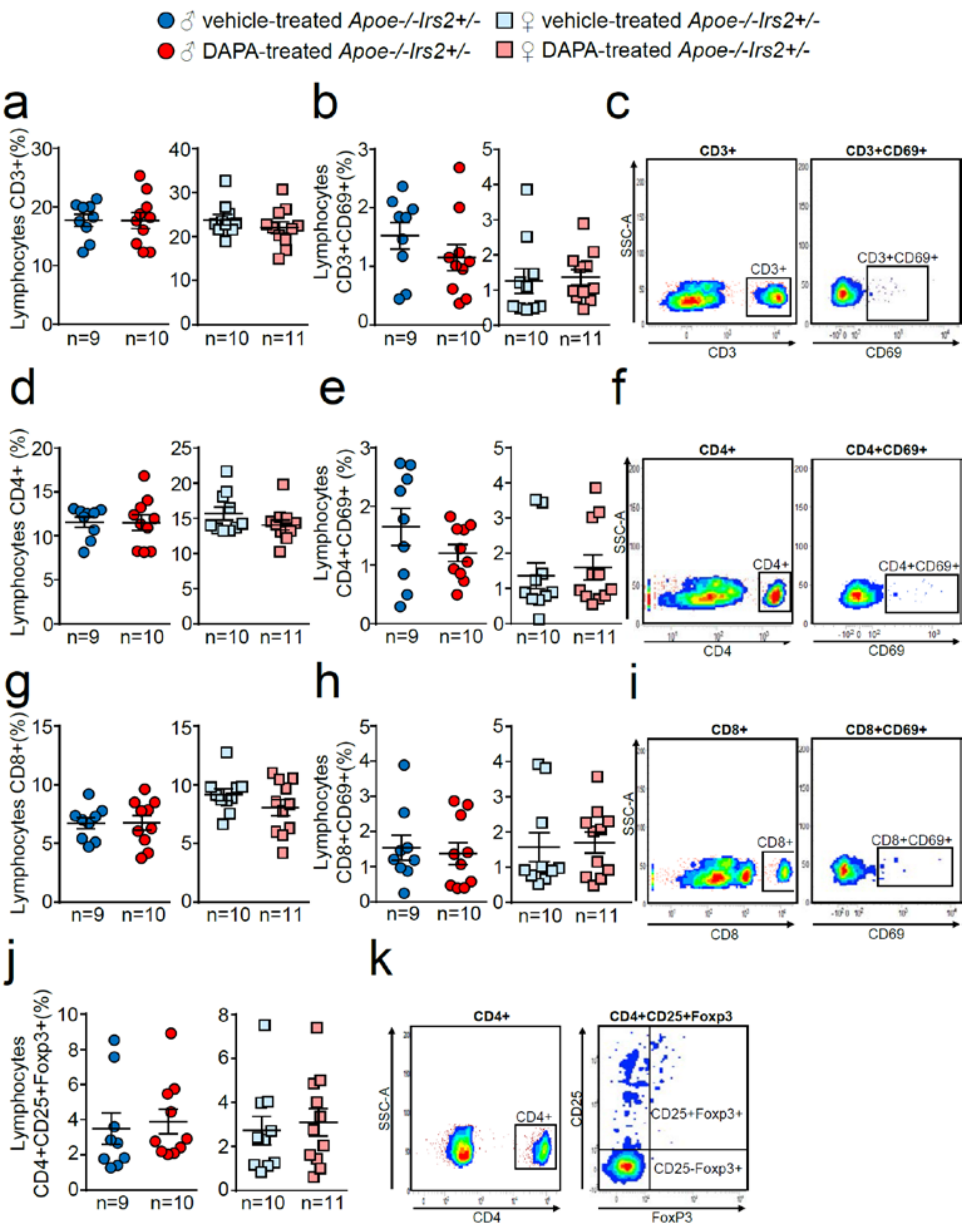

Figure S2. Analysis of circulating blood levels of total and subtypes of lymphocytes in male and female vehicle- and DAPA-treated *Apoe-/-Irs2+/-* mice. Circulating blood percentages of total **(a)** CD3+, **(b)** activated CD3+CD69+, **(d)** CD4+, **(e)** activated CD4+CD69+, **(g)** CD8+, and **(h)** activated CD8+CD69+ lymphocyte subsets in vehicle and DAPA-treated mice. **(j)** Levels of CD4+CD25+Foxp3+ Treg lymphocytes in circulating blood of vehicle and DAPA-treated *ApoE-/-Irs2+/-* mice. **(c,f,i,k)** Representative plots of the gating strategy used for flow cytometry analysis in the different lymphocyte subpopulations in blood samples are shown. The statistical analysis for normal distribution was D'Agostino-Pearson test and for differences were Student´s t-test (right panels in a,b,d and h,e,g and left panel in j) and Mann-Whitney U test (left panels in a,b,d and h, and right panel in j).

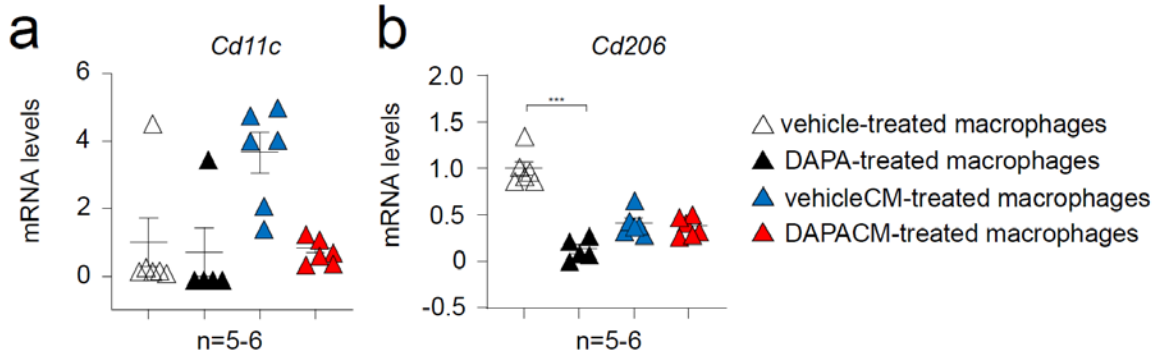

Figure S3. Effect of dapagliflozin in *Apoe-/-Irs2+/-* mouse macrophage phenotype. mRNA expression of **(a)** *Cd11c* integrin and the **(b)** *Cd206* mannose receptor in *Apoe-/-Irs2+/-* macrophages treated with vehicle (vehicle-treated), 1μM of dapagliflozin (DAPA-treated), conditioned media containing 10% of plasma from vehicle-treated mice (vehicleCM), or conditioned media containing 10% of plasma from DAPA-treated mice (DAPACM). The statistical analysis for normal distribution was Saphiro-Wilk test and for differences was Kruskal-Wallis followed by Dunn's multiple comparison test.***p<0.001.
